# Vancomycin does not affect the enzymatic activities of purified VanS_A_

**DOI:** 10.1371/journal.pone.0210627

**Published:** 2019-01-24

**Authors:** Elizabeth C. Upton, Lina J. Maciunas, Patrick J. Loll

**Affiliations:** Department of Biochemistry and Molecular Biology, Drexel University College of Medicine, Philadelphia, Pennsylvania, United States of America; Hungarian Academy of Sciences, HUNGARY

## Abstract

VanS is a membrane-bound sensor histidine kinase responsible for sensing vancomycin and activating transcription of vancomycin-resistance genes. In the presence of vancomycin, VanS phosphorylates the transcription factor VanR, converting it to its transcriptionally active form. In the absence of vancomycin, VanS dephosphorylates VanR, thereby maintaining it in a transcriptionally inactive state. To date, the mechanistic details of how vancomycin modulates VanS activity have remained elusive. We have therefore studied these details in an *in vitro* system, using the full-length VanS and VanR proteins responsible for type-A vancomycin resistance in enterococci. Both detergent- and amphipol-solubilized VanS_A_ display all the enzymatic activities expected for a sensor histidine kinase, with amphipol reconstitution providing a marked boost in overall activity relative to detergent solubilization. A putative constitutively activated VanS_A_ mutant (T168K) was constructed and purified, and was found to exhibit the expected reduction in phosphatase activity, providing confidence that detergent-solubilized VanS_A_ behaves in a physiologically relevant manner. In both detergent and amphipol solutions, VanS_A_’s enzymatic activities were found to be insensitive to vancomycin, even at levels many times higher than the antibiotic’s minimum inhibitory concentration. This result argues against direct activation of VanS_A_ via formation of a binary antibiotic-kinase complex, suggesting instead that either additional factors are required to form a functional signaling complex, or that activation does not require direct interaction with the antibiotic.

## Introduction

Enterococci are commensal bacteria normally found in the human gastrointestinal system; however, they can also cause serious and potentially fatal systemic infections such as endocarditis and bacteremia [1]. Enterococcal infections have long been treated with vancomycin, but now the rise of vancomycin-resistant enterococci (VRE) threatens the utility of the antibiotic and presents a significant public health threat [2]. VRE have acquired vancomycin-resistance genes carried on transmissible DNA elements [3]. Different phenotypic variants of VRE are known, which differ in the level of resistance conferred; of these, A-type resistance is responsible for the majority of VRE encountered in humans [4]. However, all the known resistance phenotypes share certain common properties. All produce enzymes that remodel the cell wall in order to alter vancomycin’s target; in the case of VanA, this remodeling reduces vancomycin’s affinity for its target ~1,000-fold [5–7]. All resistance operons also encode VanS and VanR, two proteins that function together to control the expression of the other resistance genes, ensuring that the remodeling enzymes are expressed only in the presence of vancomycin.

This report focuses on the VanS and VanR proteins associated with A-type resistance (herein denoted VanS_A_ and VanR_A_). VanS_A_ and VanR_A_ form a two-component system that modulates the expression of vancomycin resistance genes in response to antibiotic challenge. Two-component systems are ubiquitous in bacteria and plants [8–10] and help shape an organism’s response to signals in its environment. Two-component systems comprise a sensor and a response regulator; the former recognizes and transduces the signal, while the latter translates this signal into some cellular response. In the VanA two-component system, VanS_A_ is a histidine kinase that serves as the sensor, and VanR_A_ is a transcription factor that serves as the response regulator [11]. In the presence of vancomycin, VanS_A_ autophosphorylates on a conserved histidine residue, and then transfers this phosphoryl group to an aspartate on VanR_A_ [12, 13]. The phosphorylated VanR_A_ is the active form of the transcription factor, which binds to the *van* promoters to activate transcription of the vancomycin-resistance genes [14]. In the absence of vancomycin, VanS_A_ dephosphorylates VanR_A_, thereby maintaining it in its inactive state [11, 15]. Hence, three distinct catalytic activities are associated with VanS_A_: autophosphorylation, phosphotransfer, and dephosphorylation.

Although the broad outline of VanR-VanS functioning is known, many details remain elusive, including the mechanism by which VanS senses vancomycin. Two main models have been advanced to explain this process. The first posits a direct molecular interaction between vancomycin and the periplasmic domain of VanS, inducing a conformational change in the protein that leads to stimulation of autophosphorylation [16, 17]. The second model suggests that, rather than detecting the antibiotic directly, VanS instead senses some downstream consequence of vancomycin action, for example accumulation of a biosynthetic precursor, or a change in membrane fluidity caused by disruption of the cell wall [18–21]. Evidence is available to support both models, and there is currently not a consensus as to which (if either) is correct, or indeed if all VanS proteins even function by the same mechanism. Given the clinical importance of VRE, developing a better understanding of how VanS_A_ senses vancomycin is of paramount importance.

A purified, reconstituted VanR_A_-VanS_A_ signaling system will be of great benefit in addressing questions related to vancomycin sensing. In pioneering work from the Walsh laboratory, the cytosolic domain of VanS_A_ was previously purified [15]; however, studies of how the vancomycin signal is sensed and transduced must necessarily include the transmembrane and periplasmic domains. This imperative has prompted more recent efforts in which full-length VanS_A_ has been purified [22–25]. These studies of the full-length protein focused largely on its antibiotic binding and hydrodynamic characteristics. Now, we provide a complementary analysis of VanS_A_’s enzymatic functions. Herein we describe the purification of full-length VanS_A_, evaluate all three of its baseline enzymatic activities, assess the effect of vancomycin on these activities, and characterize its interaction with its cognate response regulator, VanR_A_. We also show that VanS_A_ activity depends strongly on the mechanism of solubilization used, consistent with models in which small rearrangements of histidine kinase transmembrane domains give rise to activation or suppression of signaling activity [26].

## Materials and methods

### Protein expression

A gene encoding full-length VanS_A_ (Uniprot Q06240) was synthesized using codon optimization for *E*. *coli* expression (Genscript) and cloned into the in-house vector pETCH [27], yielding a construct in which the sequence PGHHHHHH is appended to the C-terminus of the VanS_A_ protein. The T168K mutant of VanS_A_ was produced by a one-step PCR-based site-directed mutagenesis protocol [28]. Primers used are shown in S1 Table. To prepare an expression construct for the cytosolic domain of VanS_A_ (cVanS_A_), we used the TMHMM server [29, 30] to predict the locations of the two transmembrane helices. This analysis suggested that the cytoplasmic domain begins at residue K98. We therefore cloned the sequence corresponding to residues 98–384 into pETCH, giving a construct having the sequences MV added at the N-terminus and PGHHHHHH at the C-terminus. The codon-optimized gene for full-length VanR_A_ (Uniprot Q06239) was synthesized and subcloned into pETHSUL, [27] yielding a construct with a cleavable N-terminal 6xHis-SUMO tag. Removal of the SUMO tag with SUMO hydrolase produces a full-length VanR_A_ molecule with a single glycine residue appended to its N-terminus. The full-length VanS_A_ and VanR_A_ proteins were expressed in BL21(DE3) cells, growing cells in LB broth at 37°C to an OD_600_ of ~0.4, at which point the temperature was reduced to 18°C and IPTG was added to a final concentration of 300 μM. Cells were harvested after ~20 hours of shaking at 175 RPM. Cytosolic VanS_A_ was expressed in auto-inducing media [31] at 30°C and 225 RPM and harvested 20 hours after inoculation.

All purification steps took place at 4°C. For all constructs used (S1 Fig), purification began with cell lysis in an Emulsiflex C5 cell homogenizer at 10,000–15,000 psi in lysis buffer (50 mM Tris pH 7.7, 500 mM NaCl, 5 mM MgCl_2_). Cell lysate was clarified by centrifugation at 5,000x*g* for 15 minutes, then spun at ~117,000x*g* for 1 hour to separate the membrane fraction (high-speed pellet) from the soluble fraction (high speed supernatant). At this point, the protocols used for the different constructs diverged; specifics are given below.

Full-length VanS_A_: The membrane pellet corresponding to 6 liters’ worth of cells was homogenized in 45 mL of lysis buffer, and then 5 mL of 10% (w/v) DDM were added. The suspension was rocked at 4°C for 1 hour and then centrifuged at 117,000x*g* for 1 hour to remove unsolubilized material. The supernatant was filtered through a 0.45 micron filter and loaded onto a 1-mL HiTrap IMAC HP column (GE Life Sciences) equilibrated in IMAC A buffer (50 mM Tris pH 7.8, 500 mM NaCl, 5 mM MgCl_2_, 40 mM imidazole) plus 0.1% DDM. To exchange the protein into LDAO the column was washed with IMAC A buffer containing 10 mM LDAO. VanS_A_ was then eluted with a gradient (10 column volumes) to 100% IMAC B buffer (IMAC A+ 500 mM imidazole) + 10 mM LDAO. Fractions containing VanS_A_ were pooled and concentrated to ~500 μL and loaded onto a 1.1 cm x 50 cm Sephacryl S-300 column equilibrated with 50 mM Tris pH 7.8, 500 mM NaCl, 5 mM MgCl_2_, 10 mM LDAO. Protein fractions were pooled and concentrated to ~500 μL and dialyzed *vs*. 50 mM Tris pH 7.8, 150 mM NaCl, 5 mM MgCl_2_, 5% glycerol, 10 mM LDAO. Final detergent concentrations in the purified samples were measured using TLC [32].

cVanS_A_: The high speed supernatant from 2 liters’ worth of cells was filtered and purified by immobilized metal chromatography, as described for full-length VanS_A_ except using a 5-mL HiTrap IMAC HP column, and omitting detergents from the buffers. Fractions containing cVanS_A_ were pooled and dialyzed against IEX start buffer (50 mM Tris pH 8.0, 50 mM NaCl.) The protein was then loaded on a 5-mL HiTrap Q HP column (GE Life Sciences) and eluted with a gradient (10 column volumes) from 50 to 500 mM NaCl. Fractions containing protein were pooled, concentrated, and loaded onto an S-300 column equilibrated in SEC buffer without detergent. Finally, the protein eluting from the size exclusion column was concentrated to >10 mg/mL and dialyzed into 50 mM Tris pH 7.8, 100 mM NaCl, 5 mM MgCl_2_, 5% glycerol.

VanR_A_: The high-speed supernatant was filtered and loaded onto a 5-mL HiTrap IMAC HP column equilibrated in IMAC A buffer. Fractions containing SUMO-VanR_A_ were pooled, concentrated, and dialyzed overnight against IMAC A buffer. 200 μg of the SUMO hydrolase dtUD1 [27] was added to the dialysis bag to cleave the 6xHis-SUMO tag. The dialyzed material was passed over the same HiTrap IMAC HP column, which had been re-equilibrated in IMAC A buffer. The flow-through fractions containing VanR_A_ were pooled, concentrated, and dialyzed against 2 L IEX start buffer. The VanR_A_ was then purified by ion-exchange chromatography, using a 5-mL HiTrap Q HP column as described above. The purified VanR_A_ peak was finally dialyzed against 50 mM Tris pH 7.8, 50 mM NaCl, 5 mM MgCl_2_, and 5% glycerol.

PhoR: The gene encoding the cytosolic portion of PhoR (residues N166-D431) was amplified from *E*. *coli* strain BL21(DE3) and subcloned into the in-house vector pETHM3c. The resulting construct encodes an N-terminally His_6_-tagged MBP protein connected at its C-terminus to PhoR, via a linker containing the recognition site for the rhinovirus 3c protease. The expression vector was introduced into BL21(DE3) cells. 2-mL overnight cultures were inoculated into 1 L LB medium and grown at 37° to OD_600_ ~0.4–0.6. Expression was induced with 300 μM IPTG for 2.5 hours. The supernatant from the high-speed centrifugation was filtered and purified by immobilized-metal affinity chromatography as described for other constructs, using a 5-mL HiTrap IMAC HP column. Fractions containing the MBP-PhoR fusion protein were concentrated and dialyzed overnight vs. IEX start buffer and then purified using a 5-mL HiTrap Q HP column, as described for cVanS_A_. Fractions from this step were directly loaded on a 1.0 cm x 5.0 cm amylose column (NEB Amylose Resin E8021L) equilibrated in 50 mM Tris pH 8.0, 500 mM NaCl. After washing, the bound protein was eluted with 10 mM maltose in the same buffer. The protein was finally dialyzed into 50 mM Tris pH 8.0, 50 mM NaCl, and 10% glycerol.

### Differential filtration

The differential filtration assay was performed as described by Vergis *et al*. [33], using the Analytic Selector kit (Anatrace). Briefy, 500 μg of full-length VanS_A_ was captured using a 20% slurry of His-bind resin (Millipore) in wash buffer (50 mM Tris, 500 mM NaCl, 40 mM imidazole pH 7.7) + 0.1% DDM. The slurry was dispensed into each well of a 0.2 μm filter plate, after which the beads were washed with wash buffer + 0.1% DDM, and then detergent-exchanged into the new detergents from the Analytic Selector kit. The protein was eluted from the His-bind beads and then applied directly to either a small (100 kDa) or large (300 kDa) MWCO filter plate. The eluates from these filtrations were analyzed by dot blot using an anti-6xHis antibody (Proteintech # HRP 66005); blots were scanned using a LI-COR Odyssey-FC imaging system.

### Amphipol reconstitution

Purified, concentrated VanS_A_ protein (approx. 150 uM) containing approximately 120 mM LDAO was diluted to 22 uM (1 mg/mL) with 50 mM Tris pH 7.4, 50 mM KCl. An aliquot of a 10% w/v solution of amphipol A8-35 (Anatrace) was added to give a 1:1 mass ratio of protein to amphipol [34], and the solution was incubated on ice for 30 minutes. A quantity of washed BioBeads (BioRad) was added equivalent to 20x the estimated mass of LDAO present, and the solution was mixed at 4° for 2 hours. Pilot ultracentrifugation experiments were used to determine that this treatment gave 100% recovery of the VanS_A_ protein in the supernatant. Once this was confirmed, subsequent experiments omitted the ultracentrifugation step. After detergent removal with the BioBeads, the material was injected onto a Superdex 200 Increase 10/300 GL column (GE Healthcare) equilibrated with 20 mM Tris-Cl pH 7.4, 150 mM NaCl, and the peak corresponding to active VanS_A_ was collected and used for enzymatic assays. A second, later-eluting peak was also observed, but showed substantially lower activity. The nature of this second species was not investigated further, but we did note that the second peak showed almost no dimer band on SDS-PAGE gels, suggesting it might containing VanS_A_ monomers. Addition of 1% C12E8 to the protein solution prior to amphipol reconstitution decreased the relative size of the second peak.

### Autophosphorylation

Autophosphorylation was detected as described [35], using the bio-orthogonal ATP analog ATPγS to produce a thiophosphorylated protein. This protein was then treated with the alkylating agent *p*-nitrobenzyl mesolate (PNBM), and the resulting PNBM-derivatized thiophosphate epitope was identified immunochemically. Briefly, 1 mM ATPγS was added to 15 μM of the histidine kinase in a 15 μL volume using reaction buffer (50 mM Tris pH 7.4, 50 mM KCl, 10 mM MgCl_2_; for full-length VanS_A_ 9 mM C12E8 was also included). After incubation at room temperature for the desired length of time, the reaction was quenched with 3 μL of 500 mM EDTA, pH 8.0. Then, 1 μL of 50 mM PNBM in 100% DMSO was added and incubated at room temperature for 1 hour, after which 4 μL of 6x sample buffer were added to each reaction before SDS-PAGE. After electrophoresis, the labeled proteins were transferred to a nitrocellulose membrane and blocked with 5% nonfat dry milk in 1x TBST (20 mM Tris, 500 mM NaCl pH 8.0) for 1 hour at room temperature. The membranes were then rocked overnight at 4°C with a 1:5000 dilution of anti-phosphothioester antibody (Abcam ab92570) in 1% milk. The membrane was washed 3x10 mins with TBST, and then incubated for 1 hour at room temperature with a 1:1000 dilution of goat anti-rabbit secondary antibody (Jackson ImmunoResearch HRP-GARIgG 111-035-003) in 1% milk. Finally, the membrane was washed 3x10 mins with TBST before reaction with peroxidase substrate solution for enhanced chemiluminescence (Pierce 32209) and visualized with film. ImageJ was used for quantification and background subtraction [36]. In the case of the full-length VanS_A_ both the monomer and dimer bands were included in the quantification. Background-corrected intensities were normalized to the intensity of the 20-minute time point (corresponding to the maximum intensity observed in the course of the experiment).

### Phosphotransfer

Phosphotransfer to VanR_A_ was measured using a variation on the autophosphorylation protocol described above. Prior to the EDTA quenching step, 30 μM VanR_A_ was added to 20 μM full-length VanS_A_ (± 50 μM vancomycin) or 0.66 μM cVanS_A_. This VanR_A_ was pre-treated with iodoacetamide to reduce alkylation of cysteine residues on the protein and avoid cross-reactivity with the anti-PNBM antibody. The phosphotransfer reactions were allowed to proceed for the desired lengths of time before being quenched with EDTA. Band intensities were background-corrected and normalized to the values obtained at the longest time point (30 minutes).

### Dephosphorylation

For dephosphorylation experiments, phospho-VanR_A_ was prepared by phosphorylation with PhoR. Briefly, a 150 μl reaction containing 28 μM MBP-PhoR fusion protein was allowed to autophosphorylate in the presence of 2 mM ATP for 1 hour at room temperature in reaction buffer. The reaction was then desalted twice using Zeba Spin desalting columns (Thermo 89882) to remove nucleotide. The desalted phospho-PhoR was then diluted in a 200 μl reaction to a final concentration of 20 μM, after which 20 μM VanR_A_ was added and phosphotransfer was allowed to proceed for 2 hours at room temperature in reaction buffer. This reaction was then added to 500 μl amylose beads and incubated at room temperature for ~15 minutes to remove the MBP-PhoR. The beads were removed by filtration (0.2 micron centrifugal filter, Pall ODM02C34), and the phospho-VanR_A_ was used to set up dephosphorylation experiments. Concentrations given are nominal; no effort was made to account for dilution occurring during the desalting step or during the amylose bead treatment. Phospho-VanR_A_ was added to reaction buffer to a final concentration of ~7.5 μM, after which the histidine kinase was added to a final concentration of 7.5 μM (with or without 50 μM vancomycin). 15-μL aliquots were removed at various time points and quenched with 3 μL of 6x sample buffer. The samples were then run on a pre-cast Phos-tag gel (50 μM Phos-tag, 12.5% acrylamide; Wako) for 2–3 hours at 30 mA. Gels were stained with Coomassie Blue and photographed, after which band intensities were quantified with ImageJ.

### Surface plasmon resonance

All SPR experiments were performed on a BioRad ProteOn XPR36 at 25°C, using immobilized VanR_A_ as the ligand and full-length or cytosolic VanS_A_ constructs as mobile phase analytes. VanR_A_ was immobilized using the Rho1D4 epitope-antibody system [37]. Rho1D4 antibody was obtained from the University of British Columbia (Vancouver, Canada), and covalently attached to a GLC chip (BioRad) via amine coupling. Briefly, the chip surface was activated with a mixture of 20 mM EDC (1-ethyl-3-(3-dimethylaminopropyl)carbodiimide hydrochloride) and 5 mM sulfo-NHS (N-hydroxysulfosuccinimide) at 30 μL/min for 300 sec. 0.5 mg/mL Rho1D4 antibody was then injected on each lane at 30 μL/min for 420 seconds, yielding ~6000–7000 RU immobilized on the surface. The surface was then regenerated with 3 injections of 10 mM NaOH followed by 1% *N*-octyl-β-D-glucopyranoside (Anatrace, Maumee OH) at 100 μL/min for 18 seconds and equilibrated overnight in running buffer (50 mM BIS-TRIS pH 6.0, 150 mM NaCl, 10 mM MgCl_2_, and 0.005% Tween 20).

A VanR_A_ construct was prepared with a C-terminal 1D4 epitope tag (amino acid sequence TETSQVAPA), connected to the C-terminus of VanR_A_ by a GGGS linker. To confirm that the epitope tag does not affect protein function, phosphotransfer experiments were carried out, which showed that the 1D4-tagged VanR_A_ is a competent substrate for phosphotransfer (S5 Fig). The tagged VanR_A_ construct was diluted in running buffer to 1–25 μg/mL and passed over the chip at 50 μL/min for 30 seconds, followed by a 240-second dissociation time. This resulted in capture of 200–800 RU of the ligand. This was followed by five blank (buffer only) injections. Serial dilutions of the histidine kinases were prepared in running buffer (+ 9 mM C12E8 in the case of full-length VanS_A_), and were injected at 100 μL/min with an association time of 240 seconds and a dissociation time of 600 seconds (S4 Fig).

Equilibrium binding responses for the histidine kinases were normalized to the ligand response in each lane, using averaged response values at the end of the association phase, excluding any air bubbles (time points used to determine equilibration response values are shown as gray bars in S4 Fig). These normalized responses were then plotted as a fraction of the theoretical maximum response:

$${Response}_{max}={Response}_{ligand}\left(\frac{{mass}_{analyte}}{{mass}_{ligand}}\right)$$

Running buffer without C12E8 was used for the dissociation phase, as inclusion of C12E8 led to bubble formation. However, full-length VanS_A_ proved to be unstable in this buffer and appeared to precipitate on the chip during the dissociation phase. Hence, rather than use global fits of association and dissociation rates to derive *K*_D_ values, binding affinities were obtained by fitting a one-site binding model to the equilibrium response data.

### Fluorescence anisotropy

Cysteine modification was used to attach a fluorophore to VanR_A_ for use in anisotropy experiments. Although VanR_A_ contains multiple cysteine residues, they are not readily labeled by fluorescein maleimide (data not shown). Accordingly, site-directed mutagenesis was used to introduce an additional cysteine at the protein’s N-terminus, instead of the glycine used in the wild-type construct. A 67 μM solution of the cysteine-containing mutant in labeling buffer (50 mM Tris pH 7.8, 100 mM NaCl, 5 mM MgCl_2_, 0.05% Tween-20) was treated with 0.1 mM TCEP for 20 minutes on ice. A freshly prepared 7 mM solution of fluorescein maleimide (Thermo) in DMSO was then added to give a final fluorescein concentration of 250 μM (corresponding to a final DMSO concentration of 3.5%). The solution was incubated on ice, protected from light, for 1 hr, after which 1/10^th^ volume of 50 mM cysteine in labeling buffer was added. After an additional 20 min incubation, the solution was filtered through a 0.45 micron filter (Pall NanoSep) and loaded onto a Sephacryl S-100 HR column (1 x 45 cm) equilibrated with labeling buffer. The column was eluted isocratically at 15 cm/hr, and fractions containing VanR were pooled, concentrated to approximately 8 μM, and used within several hours for anisotropy experiments. The fluorescently-labeled VanR_A_ proved to be prone to aggregation, and so freshly prepared material was used for every experiment, and the fluorescently-tagged VanR_A_ was not tested in phosphotransfer experiments. However, the structures of the receiver domains of response regulators are conserved [38, 39], making it clear that the N-terminal labeling site is on the opposite face of the receiver domain from the site of VanR_A_ phosphorylation (Asp-53). Hence, modifications at the N-terminus are unlikely to interfere with the protein’s function.

Titration series of cVanS_A_ or MBP (negative control) were prepared in 150 μL binding reactions containing 40 nM fluorescently-labeled VanR_A_ in labeling buffer. Anisotropy measurements were conducted at room temperature using a Tecan Spark microplate reader. Samples were excited at 490 nm and emission read at 525 nm, using a 10 nm bandpass for both emission and excitation. A 510 nm dichroic mirror was used to condition the emitted signal.

## Results

### Purification of full-length VanS_A_

**Fig 1:**
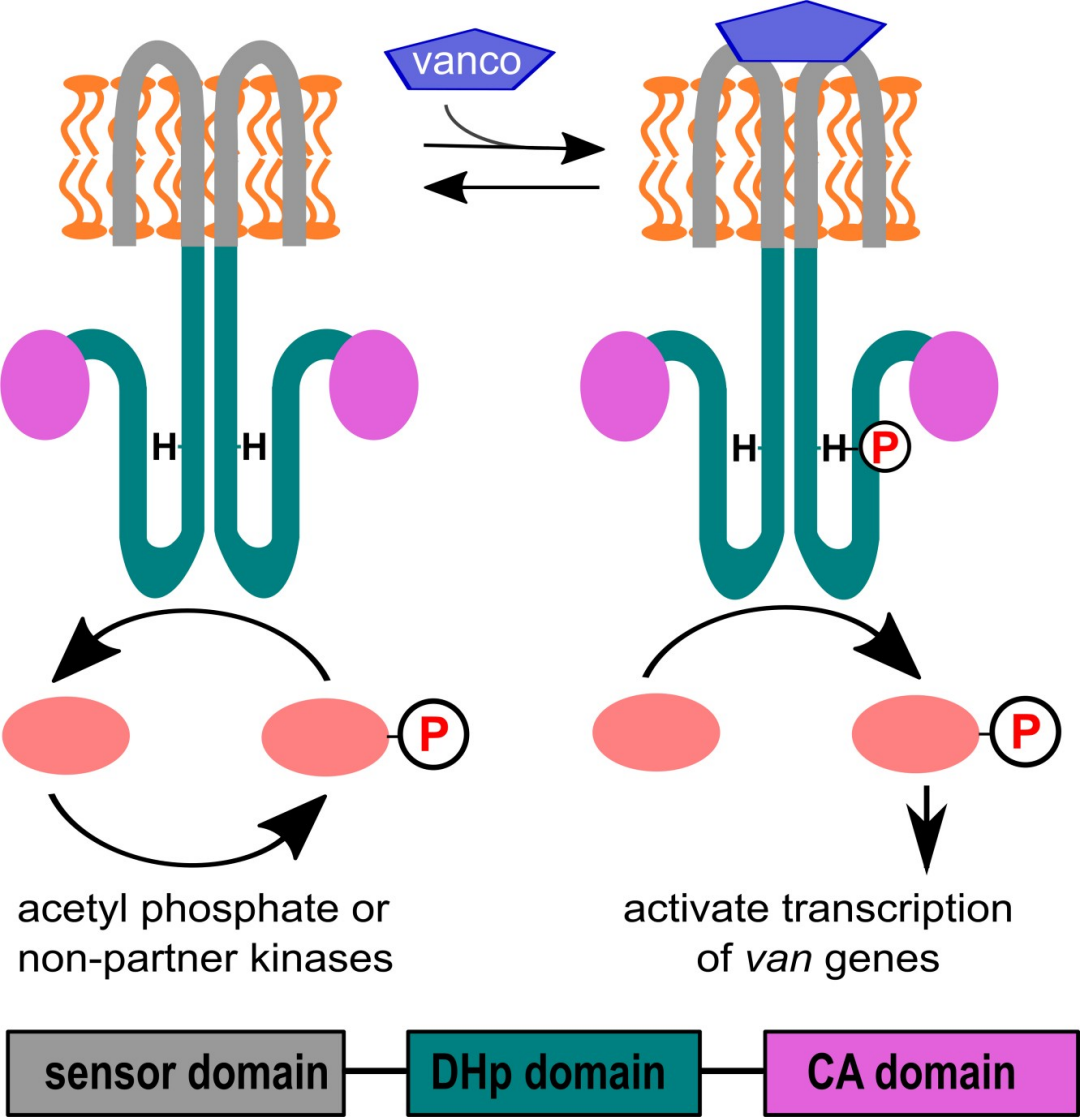
Working model for VanR_A_-VanS_A_ signaling. A schematic view is shown for the VanS_A_ protein. The protein comprises three domains: The sensor domain (gray), which includes two predicted transmembrane helices and is responsible for sensing the inducing signal; the dimerization & histidine-phosphotransfer (DHp) domain (green), which includes the conserved histidine that is the site of autophosphorylation; and the catalytic (CA) domain (magenta), which binds nucleotide triphosphate and is responsible for catalyzing histidine phosphorylation. In the absence of vancomycin, VanS_A_ acts as a phosphatase, keeping VanR_A_ in an inactive, dephosphorylated state. Vancomycin induces VanS_A_ to autophosphorylate, after which phospho-VanS_A_ transfers the phosphoryl group to VanR_A_. Finally, phospho-VanR_A_ activates transcription of the *van* resistance genes.

Biophysical probes of molecular function require access to pure, active protein. While many *in vitro* studies of the VanS_A_ protein have focused on its cytosolic fragment, the full-length protein is required to fully understand the details of signal detection and transduction (Fig 1). To complement recent studies that used purified full-length VanS_A_ to examine the protein’s binding and hydrodynamic properties [22, 23], we sought to explore the catalytic activities of VanS_A_. Therefore, we expressed and purified a full-length construct with a C-terminal His_6_-tag, using *E*. *coli* as a heterologous expression host.

**Fig 2:**
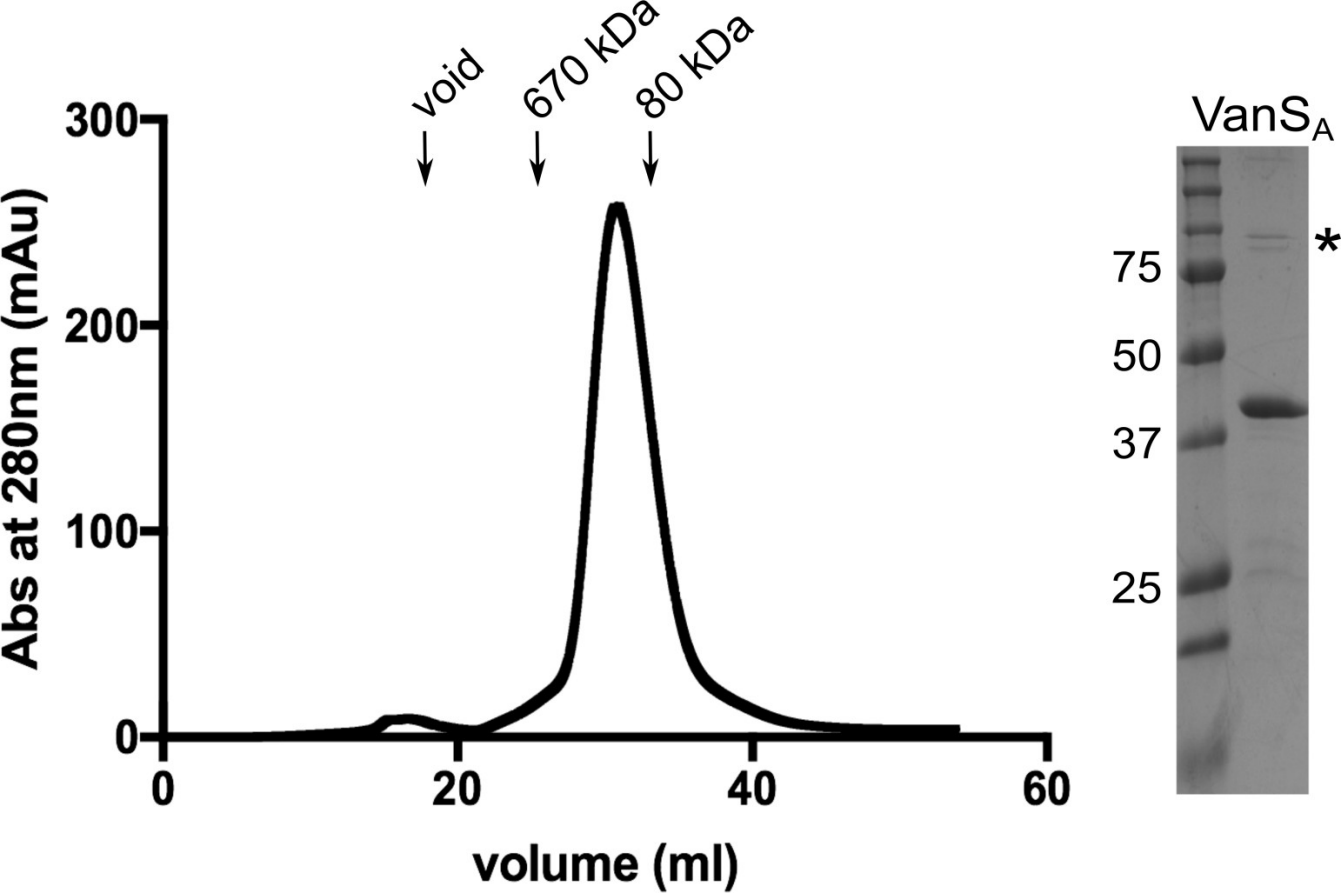
Purification of full-length VanS_A_. At left is shown the size-exclusion chromatogram corresponding to the final step of the purification of the full-length VanS_A_ protein. In LDAO, the protein elutes as a single monodisperse peak. At right is a Coomassie-stained SDS-PAGE gel showing the purified VanS_A_; the asterisk indicates the dimer band.

VanS_A_ is a membrane protein, and therefore detergents are required to remove it from the membrane and maintain it in a soluble form. A wide variety of detergents is available, and the idiosyncratic nature of proteins makes it difficult to rationally predict which will be best for any particular protein [40]. For this reason, we empirically screened different detergents for their ability to support various steps in the VanS_A_ purification. We first focused on the extraction step, in which VanS_A_ is solubilized from the membrane fraction. After extracting with different detergents, we centrifuged at high speed, and used Western blotting to detect His-tagged protein in the high-speed supernatant. In this way, we settled upon *n*-dodecyl-β-D-maltopyranoside (dodecyl maltoside, DDM) for the extraction step. We used immobilized-metal affinity chromatography (IMAC) to purify the extracted VanS_A_, and then screened 19 different detergents to find ones that allowed VanS_A_ to migrate as a monodisperse peak on a size-exclusion column. Lauryl dimethylamine oxide (LDAO) gave the best results, showing only a small aggregate peak at the void volume (Fig 2). However, we subsequently found that VanS_A_’s cognate response regulator, VanR_A_, would sometimes precipitate in the presence of LDAO, confounding efforts to study the interaction between the two proteins. At this point we revisited our choice of solubilizing agents, using a differential filtration assay to screen 96 detergents, while testing for the ability to maintain VanS_A_ in a soluble, unaggregated form [33]. Detergents that satisfied this criterion were then tested to determine if VanR_A_ remained soluble in their presence. Finally, the effect of the detergent on the autophosphorylation assay was assessed. From this effort we identified octaethylene glycol monododecyl ether (C12E8) as a detergent that maintains both proteins in a soluble form and allows for robust autophosphorylation activity. Thus, we use 1% DDM for extraction from the membrane fraction, 10 mM LDAO for IMAC, size-exclusion, and storage, and 9 mM C12E8 for activity assays.

Histidine kinases are widely considered to be obligate dimers [26]. In LDAO, VanS_A_ gives a monodisperse peak on a size exclusion column, eluting slightly earlier than the 80 kDa apotransferrin standard (Fig 2). This is consistent with the 45 kDa VanS_A_ protein behaving as an elongated dimer, as is seen for other sensor histidine kinases [23, 38, 41]; however, we note that the presence of bound detergent will increase the hydrodynamic radius of the protein and complicate estimates of size. On a denaturing SDS-PAGE gel, purified VanS_A_ migrates primarily as a monomer, but small amounts of what is evidently an SDS-resistant dimer can also be seen (S2 Fig). By the method described above, we can regularly purify 2–3 mg of well-behaved full-length VanS_A_ from 6 L of culture. In addition, we have prepared a C-terminally His-tagged construct of the cytosolic domain of VanS_A_ (cVanS_A_); this protein also shows a mobility consistent with a dimer in size exclusion chromatography (data not shown) and yields ~10–12 mg per liter of culture.

### Full-length VanS_A_ is autokinase-active

**Fig 3:**
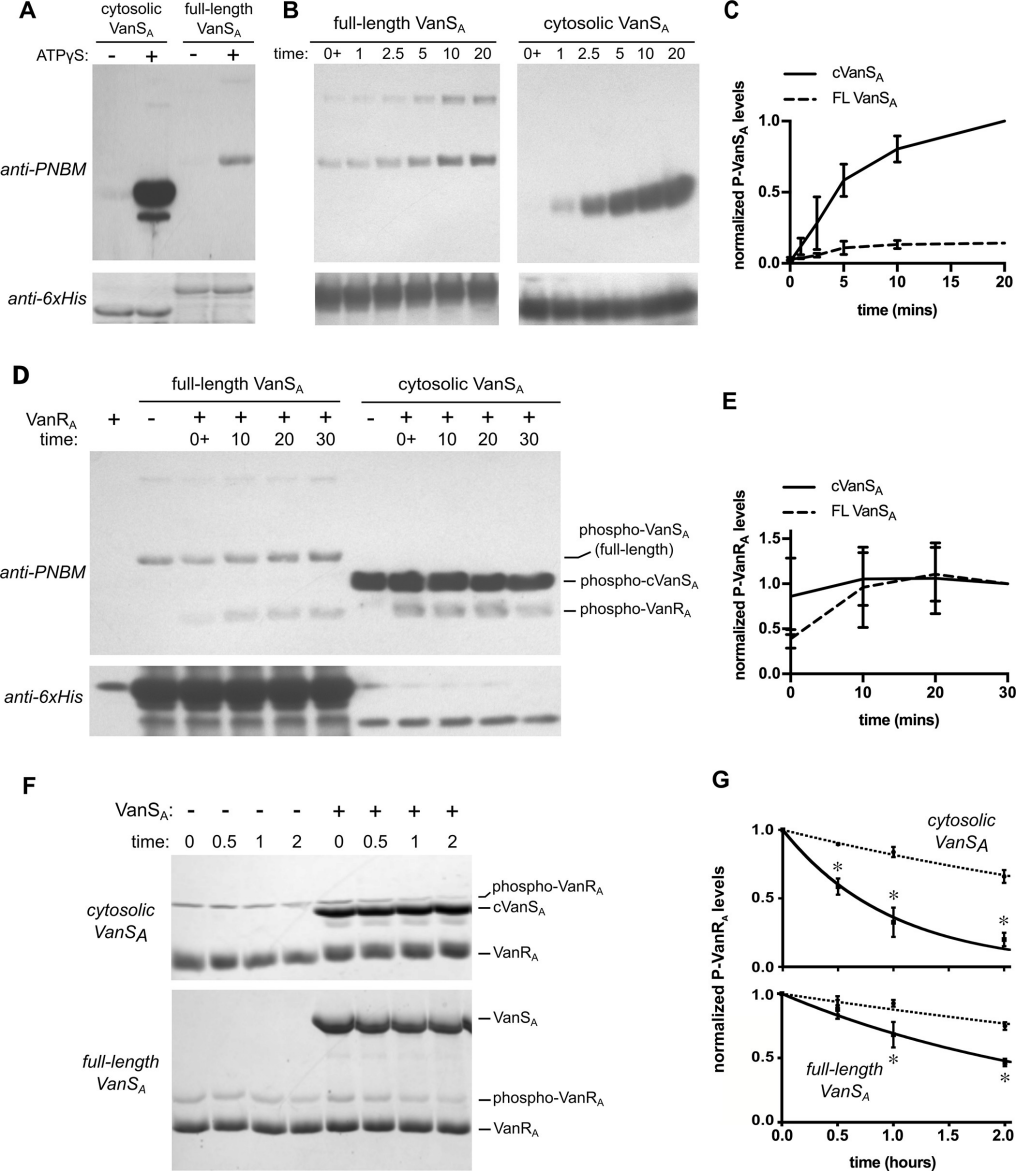
Full-length VanS_A_ displays three enzymatic activities. (**A**) Autophosphorylation of VanS_A_. Equivalent concentrations of full-length and cytosolic VanS_A_ were incubated with 1 mM ATPγS for 20 minutes. Both proteins autophosphorylate, with the cytosolic construct showing an approximately seven-fold higher level of autophosphorylation. Phosphorylation was detected by alkylation of the thiophosphoryl group with PNBM, followed by immunodetection as described in the Methods section. After quantification, blots were stripped and re-probed with an anti-6xHis antibody to verify consistent loading levels. (**B**) Time course of phosphorylation for the full-length and cytosolic VanS_A_ constructs. The two time courses were run on the same gel, but are shown as separate panels with different exposures for optimal visualization. Representative gels are shown; the graph in panel (**C**) shows the quantification of three separate autophosphorylation experiments. Band intensities are normalized to the level of phospho-cVanS_A_ at 20 minutes. (**D**) Phosphotransfer from VanS_A_ to VanR_A_. Full-length phospho-VanS_A_ (20 μM) or phospho-cVanS_A_ (0.66 μM) were mixed with 30 μM VanR_A,_ and the reaction was quenched at the times indicated. The graph in panel (**E**) shows the quantification of three separate phosphotransfer experiments; phospho-VanR_A_ levels are normalized to the levels found at 30 minutes for each kinase. (**F**) Dephosphorylation of phospho-VanR_A_. VanS_A_ (7.5 μM) was added to phospho-VanR_A_ and aliquots were removed and quenched at the times indicated. Samples were run on Phos-tag gels to separate phosphorylated and unphosphorylated VanR_A_ and stained with Coomassie Brilliant Blue. Experiments containing VanR_A_ alone (i.e., no added VanS_A_) are used to determine the intrinsic decay rate of phospho-VanR_A_. Panel (**G**) shows the averages of at least three such experiments, with phospho-VanR_A_ amounts being normalized to their starting levels. The asterisks indicate time points for which phospho-protein levels are significantly different (*p* < 0.01) between the VanR_A_-alone experiments (dashed lines) and those containing either full-length VanS_A_ or cVanS_A_ (solid lines). The lines shown represent fits of exponential decay curves to the data.

Three activities are typically associated with a sensor histidine kinase: autophosphorylation, phosphotransfer to the cognate response regulator, and dephosphorylation of the response regulator. While all three of these activities have been demonstrated for an MBP-cytosolic VanS_A_ fusion protein [15], little information is available in the literature about the activities of the full-length protein [22, 23, 25]. We began by probing the autokinase activities of our VanS_A_ constructs. Using a non-radioactive autophosphorylation assay [35], we showed that both cVanS_A_ and full-length VanS_A_ are autokinase-active (Fig 3A and 3B). The total levels of phosphorylated protein observed were substantially lower for the full-length protein than for cVanS_A_ (~7x); however, the kinetics of autophosphorylation were similar, with each construct approaching maximum autophosphorylation by 20 minutes (Fig 3B and 3C).

### Phosphotransfer from full-length VanS_A_ to VanR_A_

After autophosphorylation, the next step in the signaling process is transfer of the phosphoryl group from the histidine kinase to the response regulator. In order to determine if our cVanS_A_ and full-length VanS_A_ proteins were able to participate in phosphotransfer to VanR_A_, we performed a variation on the autophosphorylation assay described above (Fig 3D and 3E). Both cVanS_A_ and full-length VanS_A_ were found to readily transfer phosphoryl groups to VanR_A_. For cVanS_A_ the rate of phosphotransfer is very fast, with maximum transfer being achieved within a few seconds, while for full-length VanS_A_, phosphotransfer is slower, with maximum transfer being observed within 10 minutes.

### Full-length VanS_A_ dephosphorylates phospho-VanR_A_

Finally we assessed the ability of our VanS_A_ constructs to dephosphorylate phospho-VanR_A_. These assays require preparation of phosphorylated VanR_A_, followed by initiation of dephosphorylation by addition of the histidine kinase; they are complicated by the intrinsic lability of the response regulator’s phospho-aspartate moiety [42]. We chose to use a non-radioactive Phos-tag assay to measure the half-life of phosphorylated VanR_A_ in the presence and absence of VanS_A_ [42–44]. Phospho-VanR_A_ was generated using a non-cognate histidine kinase, *E*. *coli* PhoR. PhoR has previously been shown to promiscuously phosphorylate VanR_A_ in an intracellular *E*. *coli* system [45], and the purified cytosolic domain of PhoR proved to efficiently transfer a phosphoryl group to the VanR_A_ *in vitro*. After phosphorylating the response regulator, we removed the PhoR and monitored decay of phospho-VanR_A_ over time. We found that the intrinsic half-life of phospho-VanR_A_ (reflecting its decay rate in the absence of any added VanS_A_ protein) was acutely sensitive to temperature; at the ambient temperature in our laboratory (which typically falls between 20 and 25° C), the measured half-life was approximately 3.5 hours (Fig 3), slightly less than half the value reported by Wright *et al*. under somewhat different conditions [15]. Addition of either cVanS_A_ or full-length VanS_A_ significantly hastened the breakdown of the phospho-VanR_A,_ reflecting active dephosphorylation. Under these assay conditions, the full-length protein was less efficient than the cytosolic construct at catalyzing dephosphorylation.

### VanS_A_ has low micromolar affinity for VanR_A_

In some two-component systems, sensor histidine kinases have been reported to bind specifically to their cognate response regulators [46]. To assess the interaction between VanS_A_ and VanR_A_, we used surface plasmon resonance. We immobilized VanR_A_ via a C-terminal epitope tag and used the full-length and cytosolic histidine kinases as analytes in the mobile phase. Both constructs bound to VanR_A_ with dissociation constants (*K*_D_) in the low micromolar range, consistent with the affinities found for other histidine kinase-response regulator pairs [46–53] (Table 1). Full-length VanS_A_ was seen to bind VanR_A_ ~3x more tightly than the isolated cytosolic domain. When the cytosolic VanS_A_ was allowed to autophosphorylate immediately before the binding experiment, a modest increase in affinity was observed, suggesting that VanR_A_ may bind the phospho-histidine form of VanS_A_ more tightly; however, it must be noted that the cVanS_A_ was not expected to be fully phosphorylated in this experiment, and the amount of phosphorylated material may also change over the course of the analysis, making interpretation of this result difficult.

**Table 1:** Binding affinities for VanR_A_ and VanS_A_ constructs.

Histidine kinase	*K*_D_ (μM)
Cytosolic VanS_A_	6.8 ± 1.4
Phospho-cytosolic VanS_A_	2.5 ± 0.8
Full-length VanS_A_	1.9 ± 0.7

To confirm the interaction between VanS_A_ and VanR_A_, we employed fluorescence anisotropy as an orthogonal method. Only the cytosolic VanS_A_ construct was used, to avoid non-specific interactions between hydrophobic fluorophore and detergent micelles. These experiments confirmed a specific binding interaction between cytosolic VanS_A_ and VanR_A_ (S4 Fig), giving a slightly lower *K*_D_ value than that derived from the SPR experiments. The reason for the difference in *K*_D_ estimates is not yet clear, but both methods consistently demonstrate specific binding between the purified sensor histidine kinase and its cognate partner.

### Vancomycin does not alter the activity of full-length VanS_A_

**Fig 4:**
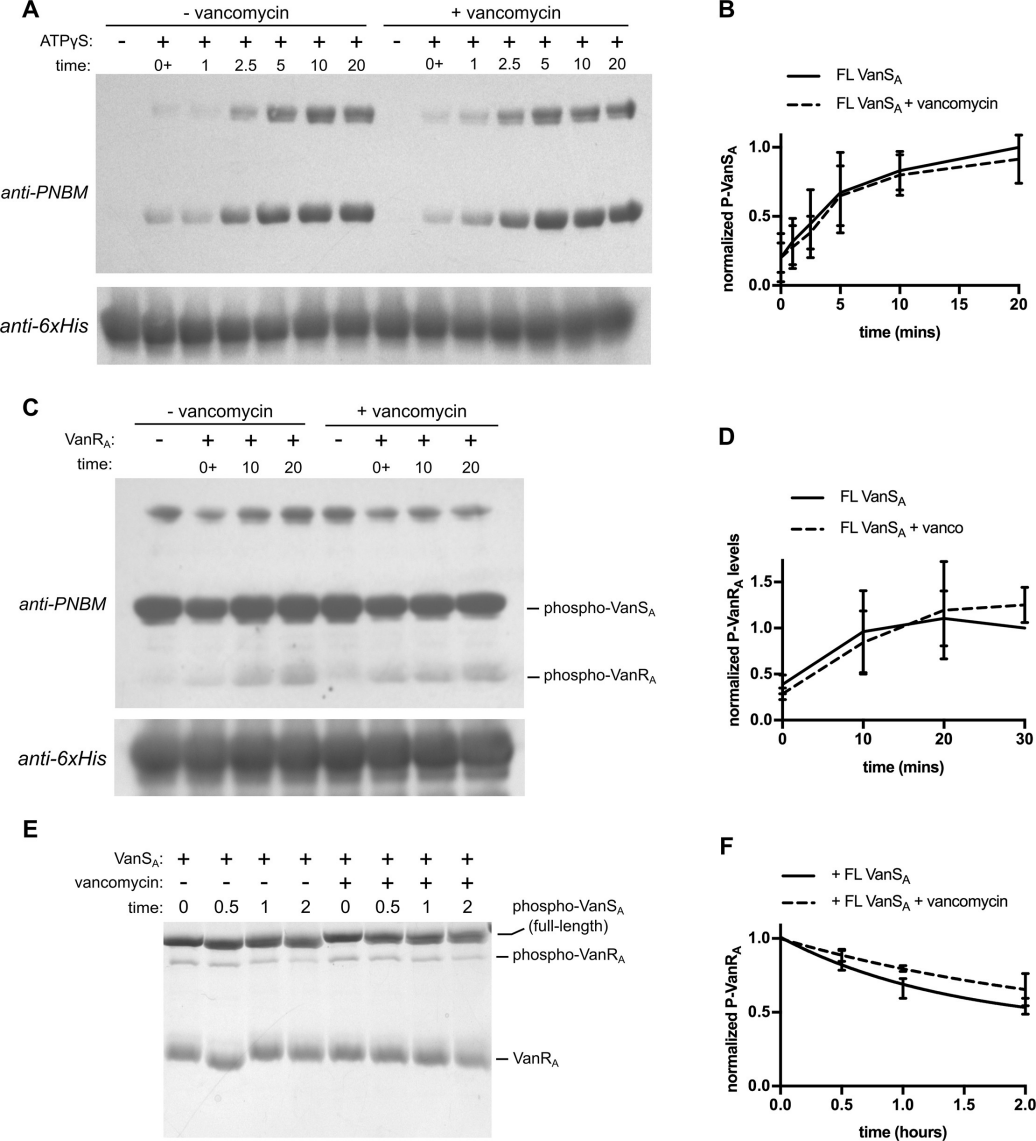
Vancomycin does not affect activity of full-length VanS_A_. (**A**) Autophosphorylation activity of VanS_A_ ± 50 μM vancomycin. Protein (15 μM) was incubated with or without 1 mM ATPγS for the times indicated. Panel (**B**) shows the quantification, displaying phospho-VanS_A_ band intensities normalized to the level attained at 20 minutes in the absence of vancomycin. (**C**) Phosphotransfer activity. 20 μM phospho-VanS_A_ was mixed with 30 μM VanR_A_ (± 50 μM vancomycin) and quenched at the times indicated. Panel (**D**) shows the quantification; phospho-VanR_A_ band intensities are normalized to the level attained in the 30-min reaction in the absence of vancomycin. (**E**) Dephosphorylation activity. 7.5 μM VanS_A_ (± 50 μM vancomycin) was added to ~7.5 μM phospho-VanR_A_, and aliquots were removed and quenched at the times indicated. Panel (**F**) shows the quantification with amounts of phospho-VanR_A_ normalized to their starting values. Fitting decay curves to these data gives slightly different estimates for the phospho-VanR_A_ half-life in the presence and absence of vancomycin; however, a t-test indicates that at each time point, the values with and without antibiotic are not significantly different. Error bars in the quantification plots represent standard deviations of at least three independent experiments.

If VanS_A_ directly recognizes the vancomycin molecule, it makes sense that antibiotic binding should function as a trigger to alter the catalytic properties of the enzyme. To probe whether vancomycin influences any of the enzymatic activities of VanS_A_, we assayed for autophosphorylation, phosphotransfer, and dephosphorylation in the presence of 50 μM vancomycin, which is ~50x the typical minimum inhibitory concentration (MIC) seen for vancomycin-sensitive enterococcal strains [54, 55]. In none of these assays did vancomycin significantly alter the enzymatic activity of full-length VanS_A_ (Fig 4); in fact, no effect was seen even when vancomycin concentration was increased to 200 μM (data not shown).

### Consequences of a potential activating mutation

While detergent is necessary to extract the protein from the membrane and maintain it in soluble form, its use complicates the analysis of the protein’s activity, as noted above. In light of our observation that vancomycin fails to affect VanS_A_’s catalytic activities, we wished to be certain that the detergent was not altering the fundamental properties of the protein. To test for this, we probed whether detergent would alter the behavior of a VanS_A_ mutant expected to be constitutively active, reasoning that if detergent is causing unphysiological behavior, it will likely perturb the constitutive behavior of the mutant.

**Fig 5:**
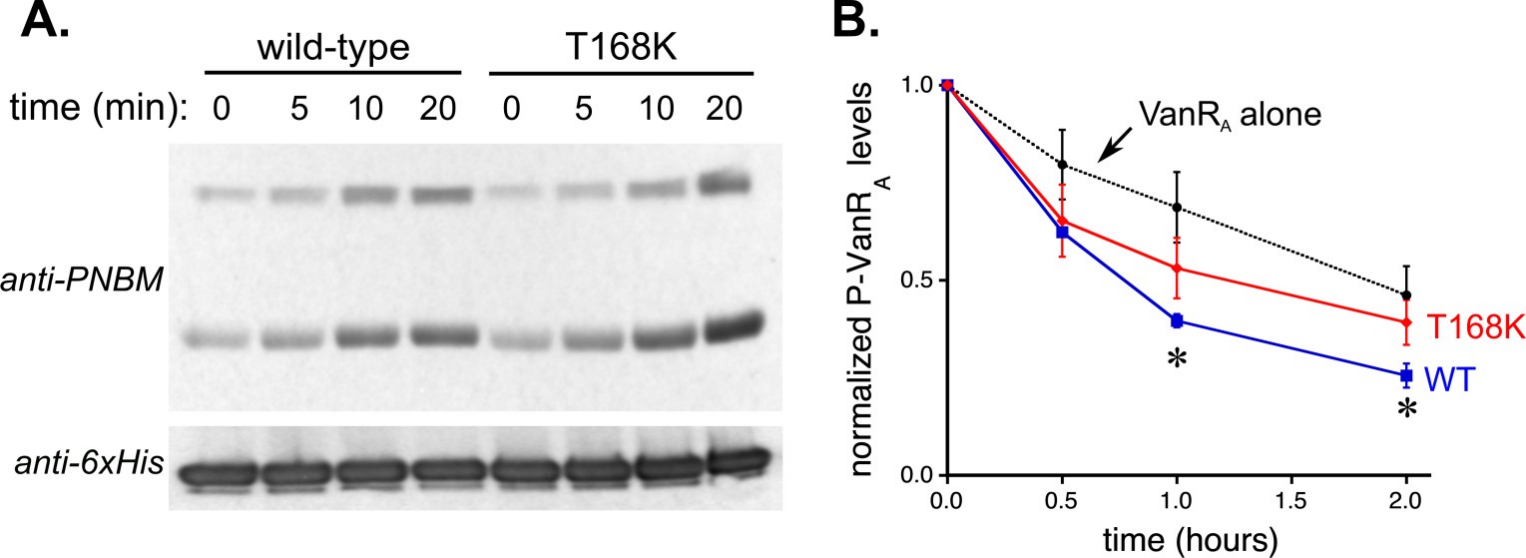
Autophosphorylation and phosphatase activities of the T168K mutant. (A) Representative autophosphorylation assays for wild-type and mutant VanS_A_. No significant difference is seen in the autokinase activities of the two proteins. (B) Dephosphorylation activities of wild-type (black solid line) and mutant VanS_A_ (red). Asterisks indicate time points for which levels of phospho-VanR_A_ are significantly different (*p* < 0.05) for the two proteins. At all time points, the phospho-VanR_A_ levels seen with the mutant protein are not significantly different from those seen with VanR_A_ alone (black dotted line).

We were unable to find documented examples of specific mutations in VanS_A_ that confer a constitutive phenotype. However, mutations associated with such a phenotype have been found in related VanS proteins. For example, in B-type VRE strains the T237K and T237M mutations have both been found in constitutively active variants [56, 57]. In the VanS_B_ protein, Thr-237 is four residues (one turn of a helix) downstream of the site of histidine phosphorylation. A similar mutation has been described in D-type strains, where a mutation in the corresponding residue (T170I) was found in a constitutive strain [58]. More generally, it appears that the presence of a threonine or asparagine four residues downstream of the phosphorylated histidine is an important determinant of phosphatase activity in histidine kinases [59, 60]. Accordingly, we chose to mutate the corresponding threonine residue in VanS_A_, Thr-168, to lysine, mimicking the T237K mutation in VanS_B_. When purified, the detergent-solubilized T168K VanS_A_ mutant exhibited an autophosphorylation activity comparable to that of the wild-type protein, but its phosphatase activity was significantly impaired (Fig 5). Thus, it is clear that the T168K substitution perturbs the balance between autophosphorylation and phosphatase activities of the purified protein, consistent with the expected constitutive activity. Importantly, this observation increases confidence that detergent is not eliciting new or unphysiological behaviors in VanS_A_.

### Amphipol trapping of VanS_A_

**Fig 6:**
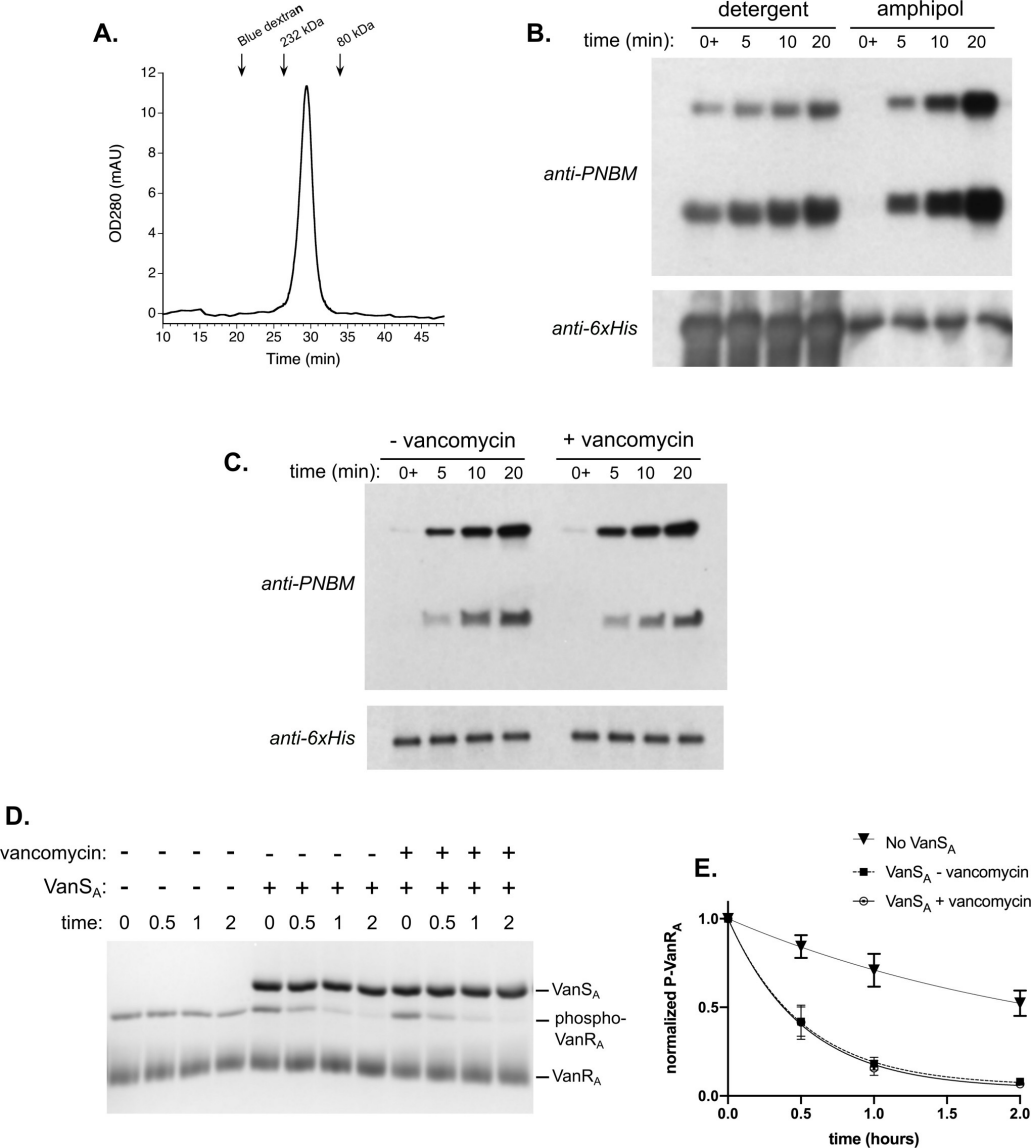
Reconstitution of full-length VanS_A_ with the amphipol A8-35. (A) Size-exclusion chromatogram of amphipol-reconstituted VanS_A_, demonstrating the presence of a single soluble and homogeneous species. (B) Comparison of autophosphorylation activity for VanS_A_ in detergent solution vs. amphipols. The amphipol-reconstituted species exhibits substantially higher enzymatic activity. This is demonstrated in the anti-6xHis loading control, which shows that small amounts of the protein-plus-amphipol solution give essentially equivalent activity to much larger amounts of the detergent-solubilized protein. (C) 50 μM vancomycin has no effect on the autophosphorylation activity of amphipol-reconstituted VanS_A_. (D) & (E) 50 μM vancomycin has no effect on the phosphatase activity of amphipol-reconstituted VanS_A_. Panel (D) shows a representative Phos-tag gel, with panel (E) showing the quantification of assays involving three independent sets of measurements.

Another way to approach the question of whether detergents are affecting VanS_A_ activity is to transfer the protein to a different environment. One such environment is provided by amphipols, which are amphipathic polymers designed to wrap around a membrane protein’s hydrophobic surfaces and mask them from aqueous solvent [61]. Because each polymer makes multiple hydrophobic contacts with the protein, amphipols bind membrane proteins tightly, and exhibit a very slow rate of unbinding [34]. Accordingly, we transferred VanS_A_ from detergent solution to the most commonly used amphipol, A8-35, and removed the detergent using BioBead treatment followed by gel filtration. The resulting VanS_A_-amphipol complex migrates as a single peak on a size-exclusion column (Fig 6A), demonstrating that the protein remains stable and homogeneous even after the removal of detergent. In contrast, the same detergent removal protocol, when performed in the absence of amphipol, results in the complete precipitation of the protein (data not shown). Interestingly, transferring the protein from detergent solution to an amphipol environment significantly enhances its autophosphorylation and phosphatase activities (Fig 6B and 6D). Critically, while the basal levels for these activities increase substantially, the activities themselves remain insensitive to vancomycin in the amphipol-solubilized protein (Fig 6C).

## Discussion

Biochemical and biophysical studies benefit greatly from the ability to reconstitute functional complexes from purified components. For a signaling complex such as the VanR-VanS system, access to isolated proteins allows for the removal of complicating factors, such as the contributions of cross-talk from other cellular proteins and the effects of non-enzymatic phosphorylation by small-molecule phospho-donors. Additionally, obtaining purified full-length protein is particularly important in the case of VanS, in order to address the question of whether vancomycin binds directly to VanS to modulate its activity. We have purified the full-length VanS_A_ protein associated with type-A enterococcal resistance, and demonstrate that the detergent-solubilized VanS_A_ demonstrates all three enzymatic activities expected of a histidine kinase, namely autophosphorylation, phosphotransfer to its cognate response regulator, and dephosphorylation of the response regulator. We also describe a likely constitutively active mutant with impaired phosphatase activity, and describe a protocol for generating a detergent-free, amphipol-solubilized form of the protein, for which enzymatic activity levels are significantly enhanced to the detergent-solubilized form. It is instructive to compare the activities of these full-length VanS_A_ protein preparations to those of the isolated cytoplasmic domain, which lacks the regulatory capabilities conferred by the transmembrane sensor domain.

In detergent solutions, the full-length VanS_A_ protein exhibits lower autophosphorylation and phosphatase activities than the cytosolic domain (Fig 3A). The rate of phosphotransfer catalyzed by full-length VanS_A_ also appears slightly slower than that seen with the cytoplasmic domain (Fig 3D and 3E), but this probably reflects different starting levels of phospho-histidine—because the phospho-histidine is a substrate in the bi-molecular phosphotransfer reaction, lower levels will translate into a lower apparent phosphotransfer rate. Indeed, one expects that phosphotransfer rates should be similar for both the cytosolic and full-length constructs, since in most two-component systems, the catalytic machinery for phosphotransfer resides primarily in the response regulator, rather than in the histidine kinase [8].

To determine whether the lower activity observed with full-length VanS_A_ in detergent reflects a regulatory effect contributed by the protein’s transmembrane region, or a nonspecific detergent effect, we used amphipol A8-35 [62] as an alternative means of solubilizing the protein. In the amphipol-solubilized form, the protein regains high levels of activity, comparable to those observed for the cytosolic construct. Therefore, it does appear that detergent is suppressing the enzymatic activity of the protein. This is consistent with the published report that VanS_A_ autokinase activity is exquisitely sensitive to DDM concentration [25]. To assess whether the detergent effect reflects direct inhibition of the protein’s catalytic machinery, one can examine the effects of detergent on the activity of the cytoplasmic construct; however, such experiments do not reveal any drastic changes in activity (S3 Fig). Hence, it seems likely that the detergent effects observed are a manifestation of structural and/or dynamic changes in the protein, such as perturbations in monomer-dimer equilibrium or subtle alterations in helix packing. The reduced activity therefore indicates that the micellar environment provided by the detergent does not mimic the native membrane environment with complete fidelity.

However, while detergents may reduce the activity of VanS_A_, there is reason to believe that they are not drastically altering the intrinsic properties of the enzyme. Specifically, we have created a constitutively activated form of VanS_A_, T168K, in which we removed a conserved threonine lying one turn of a helix downstream of the phosphorylated histidine. This mutation is predicted to reduce or completely remove phosphatase activity [59], and indeed in the mutant protein the phosphatase activity is reduced to unmeasurable levels, while the autokinase activity remains unchanged.

Perhaps the most interesting results to emerge from this work speak to the question of whether vancomycin and related antibiotics are sensed directly, via a molecular interaction with VanS_A_, or indirectly, via detection of a downstream consequence of antibiotic action. We show that high concentrations of vancomycin do not alter the enzymatic properties of VanS_A_, regardless of whether the protein is solubilized in detergent or with an amphipol. We studied vancomycin concentrations ranging from ca. 50-fold to 200-fold above the MIC levels for vancomycin-sensitive enterococci. Presumably, vancomycin-resistant enterococci must sense the antibiotic and mount a defensive response well before such a toxic threshold is reached; this implies that if the antibiotic is to exert a direct effect on VanS_A_’s enzymatic function, this effect should occur well below the concentrations we have tested.

We note that our results appear to conflict with a report that vancomycin modestly increases turnover in a general ATPase assay [22]. However, the relevance of such an ATPase assay is unclear, since the turnover numbers reported indicate many rounds of ATP hydrolysis per VanS molecule per minute. Such rapid ATP turnover is inconsistent with the stable VanS histidine phosphorylation observed by us (this work) and others [15], and would represent highly unusual behavior for a sensor histidine kinase. In contrast to the general ATPase assay, the activity measurements we report reflect physiologically relevant enzymatic activities directly related to the biological function of the VanS_A_ protein. Vancomycin’s failure to alter these activities in our purified system argues against a direct sensing mechanism involving a binary antibiotic-VanS_A_ complex. Of course, it is possible that direct sensing *does* occur, but requires additional partners that are not present in our minimal system; it is also possible that a natural inhibitor of VanS_A_ is missing from our system, and its absence leads the enzyme to adopt a fully active form, incapable of further stimulation by antibiotic.

We stress that our experiments measure activity, not binding, and therefore do not specifically address the question of whether vancomycin binds directly with VanS_A_. Recent reports have shown that vancomycin can elicit spectroscopic and hydrodynamic changes in purified full-length VanS_A_ [22, 23], which are interpreted as evidence for direct binding of the antibiotic by the protein [63]. This argument suggests that vancomycin may indeed be binding VanS_A_ in our assays, even though any such binding is not reflected by a change in enzymatic activity. However, an important caveat is that the *K*_d_ values inferred from the binding experiments are approximately 70 uM, and, as discussed above, it is difficult to reconcile such high dissociation constants with the much lower MIC values seen in vancomycin-sensitive organisms.

## Conclusions

In summary, we have characterized the enzymatic properties of full-length VanS_A_, using both detergent and amphipols to solubilize this membrane-bound protein. Both forms of the protein exhibit all three of the activities expected from a sensor histidine kinase, with the amphipol-solubilized material showing significantly higher levels of activity overall. Vancomycin, the putative inducer of the VanR-VanS two-component system, does not alter the enzymatic activity of either form of the protein, suggesting that either the antibiotic does not interact directly with VanS_A_, or that additional factors are required to confer vancomycin sensing upon the protein.

## Supporting information

S1 Table Primers used to prepare expression constructs. (DOCX)

S1 Fig VanR_A_ and VanS_A_ proteins used in this study. Our full-length VanS_A_ construct contains 392 amino acids (including the C-terminal PG-6xHis tag). The cytosolic VanS_A_ construct begins at Lys-98, immediately after the second predicted transmembrane domain; it contains two residues at the N-terminus (MV) that were contributed by the vector. The VanR_A_ construct was expressed as a fusion protein with an N-terminal, 6xHis-SUMO tag. After removal of the SUMO partner, the protein contains the full VanR_A_ sequence, along with a single additional glycine residue at the N-terminus (contributed by the vector).
(TIF)

S2 Fig Electrophoresis of full-length VanS_A_ reveals a covalent, disulfide-linked dimer. In denaturing SDS-PAGE, the main band for purified VanS_A_ migrates at the expected monomer molecular weight, 45 kDa. However, a second band is consistently observed at ~90 kDa, corresponding to the molecular weight of a dimer (labeled with an asterisk in the gels shown). This upper band is labeled in both the anti-6xHis Western blot and in the anti-PNBM blot used for the autophosphorylation assay. Together, these facts point to this upper band being a VanS_A_ dimer. SDS-resistant oligomers are commonly seen for membrane proteins, but we also considered the possibility that this band might represent a disulfide-linked dimer, since VanS_A_ contains a single cysteine near the end of the second predicted transmembrane helix. The upper band withstands treatment with normal loading buffer, which contains a final concentration of 0.1 M DTT (panel **A**, left lane). However, we reasoned that the DTT may have become oxidized and lost efficacy after several freeze-thaw cycles, and therefore tested exposure to either 50 mM TCEP or fresh 5% β-mercaptoethanol for 10 minutes before adding loading buffer. Both of these treatments removed the upper band (panel **B**), indicating that it is indeed a disulfide-linked dimer.
(TIF)

S3 Fig Effect of C12E8 on enzymatic activities of cVanS_A_. (**A**) C12E8 has at most a modest effect on autophosphorylation. Upper panel shows an anti-PNBM blot labeling phosphorylated cVanS_A_ in the presence and absence of 9 mM C12E8. The anti-6xHis blot is used as a loading control. The quantitation of the autophosphorylation is shown in panel (**B**); band intensities are normalized to the intensity of the 20-minute time point for the detergent-free reaction. (**C**) Phosphotransfer is not significantly affected by the presence of C12E8. Upper panel shows an anti-PNBM blot in which both phosphorylated cVanS_A_ and VanR_A_ are labeled; lower panel shows an anti-6xHis blot serving as a loading control for His_6_-tagged cVanS_A_. (**D**) Quantitation plot for phosphotransfer reaction; band intensities for phospho-VanR_A_ are normalized to the phospho-VanR_A_ level at 30 minutes produced by cytosolic VanS_A_ in the absence of C12E8. (**E**) Effect of C12E8 on the rate of cVanS_A_-catalyzed dephosphorylation of phospho-VanR_A_. Here we show the data from the average of 3 experiments and the fitted half-life curves for dephosphorylation with and without 9 mM C12E8. A modest reduction of activity is seen at the 2-hour time point (*p* < 0.05), but not at earlier time points.
(TIF)

S4 Fig VanR_A_ binding by VanS_A_ and PhoR probed by SPR. Panels (**A**) through (**C**): At left are shown representative sensorgrams from SPR experiments using immobilized VanR_A_. The analytes used were as follows: (**A**) cVanS_A_, (**B**) full-length VanS_A_, and (**C**) autophosphorylated cVanS_A_. The corresponding normalized equilibrium response fits are shown at right. Concentrations shown are for dimers of the histidine kinases. Gray boxes in the sensograms represent the response range used to determine the equilibrium fit. (**D**) Verification of VanR_A_ binding by cVanS_A_ using fluorescence anisotropy. A representative binding curve is shown for cVanS_A_ binding to fluorescently labeled VanR_A_. The overall change in anisotropy is small, as expected for the binding of a medium-sized protein such as VanR_A_ to a medium-sized partner. However, the binding experiments yielded reproducible results with each fresh preparation of fluorescein-labeled VanR_A_. The curve shown represents a binding isotherm corresponding to a *K*_D_ values of 0.2 μM. Experiments done on different days with different preparations of fluorescently labeled VanR_A_ consistently gave *K*_D_ values in the range of 0.1 to 1.0 μM.
(TIF)

S5 Fig 1D4-tagged VanR_A_ is a competent substrate for phosphotransfer. An anti-PNBM blot shows the time course of phosphotransfer from cytosolic VanS_A_ to wild-type VanR_A_ (left) and 1D4-tagged VanR_A_ (right). For both VanR_A_ constructs, rapid phosphotransfer is observed (within the mixing time of the experiment), followed by gradual loss of signal due to the phosphatase activity of VanS_A_. The 1D4-tagged construct migrates at a slightly larger molecular weight than the wild-type VanR_A_ protein, owing to the additional 13 residues contributed by the linker and epitope tag.
(TIF)
