# Supplementary material for: Vancomycin does not affect the enzymatic activities of purified VanSA
Source: PLoS One. 2019 Jan 24;14(1):e0210627. doi: 10.1371/journal.pone.0210627 (PMC6345502; doi:10.1371/journal.pone.0210627)
Supplement: S1 Table — (DOCX) [file pone.0210627.s001.docx]

| **Primer name** | **Primer sequence** |
| --- | --- |
| VanS_A_ forward | 5’-TTTTTTTTTTCCATGGTTATCAAACTGAAAAACAAAAAAAATGAC-3’ |
| VanS_A_ reverse | 5’-TTTTTTTTTCCCGGGGGAGCGACGTTTGTCAACCAG-3’ |
| T168K forward | 5’-CATCAAAAAACCGCTGACGAGCATTATCGGTTACCTGTCTCTGCTG-3’ |
| T168K reverse | 5’-GTCAGCGGTTTTTTGATGTCATGCGCCAGATACATAACCACATC-3’ |
| cVanS_A_ forward | 5’-AAAAAAAAACCATGGTTAAATTTGCAAAATACTTCGACGAA-3’ |
| VanR_A_ forward | 5’-AGATTGGTGGCGGAATGAGCGATAAGATCCTGATTGTG-3’ |
| VanR_A_ reverse | 5’-GAGGAGAGTTTAGACATTATTTCTCAATCTTATAGCCAAC-3’ |
| PhoR forward | 5’-AAAAAAAAACCATGGTCAATCTGGTGCTCAACACCGGG-3’ |
| PhoR reverse | 5’-AAAAAAAAAGAATTCATTAATCGCTGTTTTTGGCAATTAAACGT-3’ |
