# Supplementary figures and images for: Vancomycin does not affect the enzymatic activities of purified VanSA
Source: PLoS One. 2019 Jan 24;14(1):e0210627. doi: 10.1371/journal.pone.0210627 (PMC6345502; doi:10.1371/journal.pone.0210627)

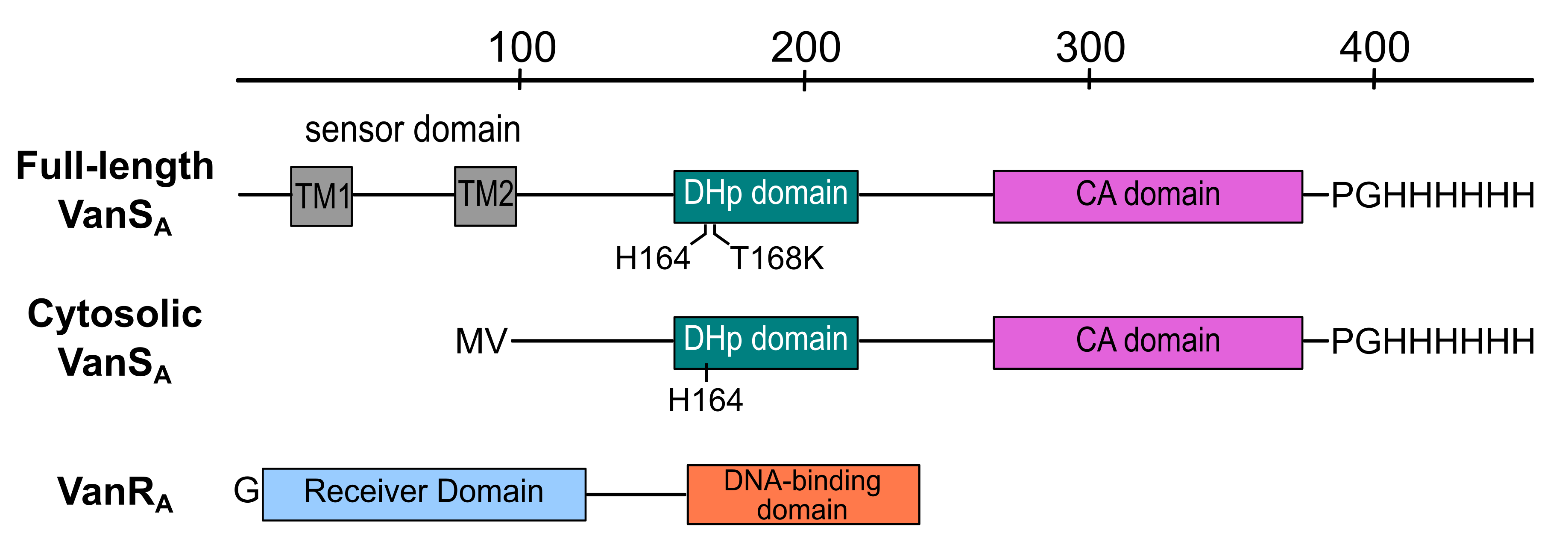

Supplement: S1 Fig — Our full-length VanSA construct contains 392 amino acids (including the C-terminal PG-6xHis tag). The cytosolic VanSA construct begins at Lys-98, immediately after the second predicted transmembrane domain; it contains two residues at the N-terminus (MV) that were contributed by the vector. The VanRA construct was expressed as a fusion protein with an N-terminal, 6xHis-SUMO tag. After removal of the SUMO partner, the protein contains the full VanRA sequence, along with a single additional glycine residue at the N-terminus (contributed by the vector). (TIF) [file pone.0210627.s002.tif]

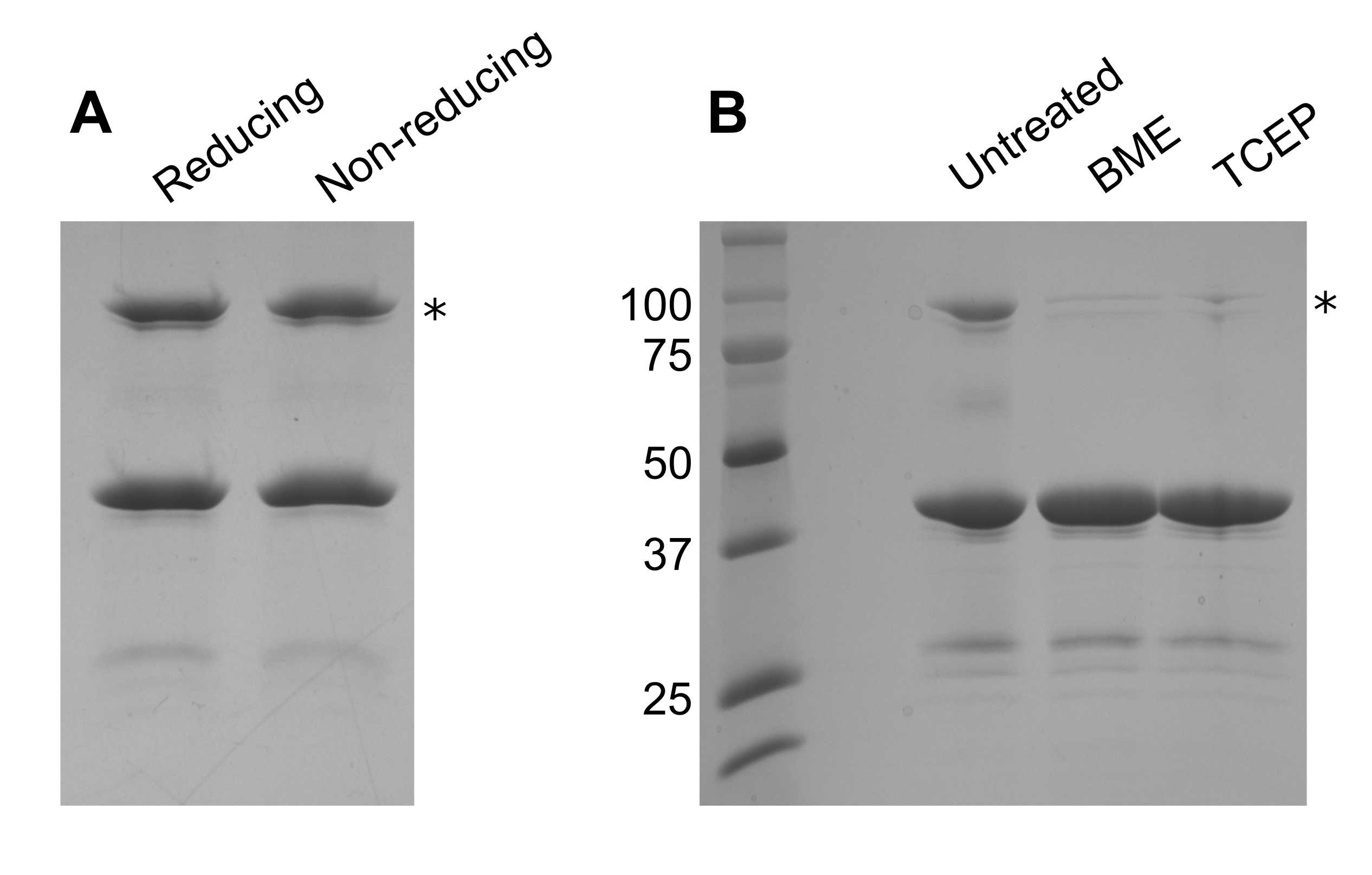

Supplement: S2 Fig — In denaturing SDS-PAGE, the main band for purified VanSA migrates at the expected monomer molecular weight, 45 kDa. However, a second band is consistently observed at ~90 kDa, corresponding to the molecular weight of a dimer (labeled with an asterisk in the gels shown). This upper band is labeled in both the anti-6xHis Western blot and in the anti-PNBM blot used for the autophosphorylation assay. Together, these facts point to this upper band being a VanSA dimer. SDS-resistant oligomers are commonly seen for membrane proteins, but we also considered the possibility that this band might represent a disulfide-linked dimer, since VanSA contains a single cysteine near the end of the second predicted transmembrane helix. The upper band withstands treatment with normal loading buffer, which contains a final concentration of 0.1 M DTT (panel A, left lane). However, we reasoned that the DTT may have become oxidized and lost efficacy after several freeze-thaw cycles, and therefore tested exposure to either 50 mM TCEP or fresh 5% β-mercaptoethanol for 10 minutes before adding loading buffer. Both of these treatments removed the upper band (panel B), indicating that it is indeed a disulfide-linked dimer. (TIF) [file pone.0210627.s003.tif]

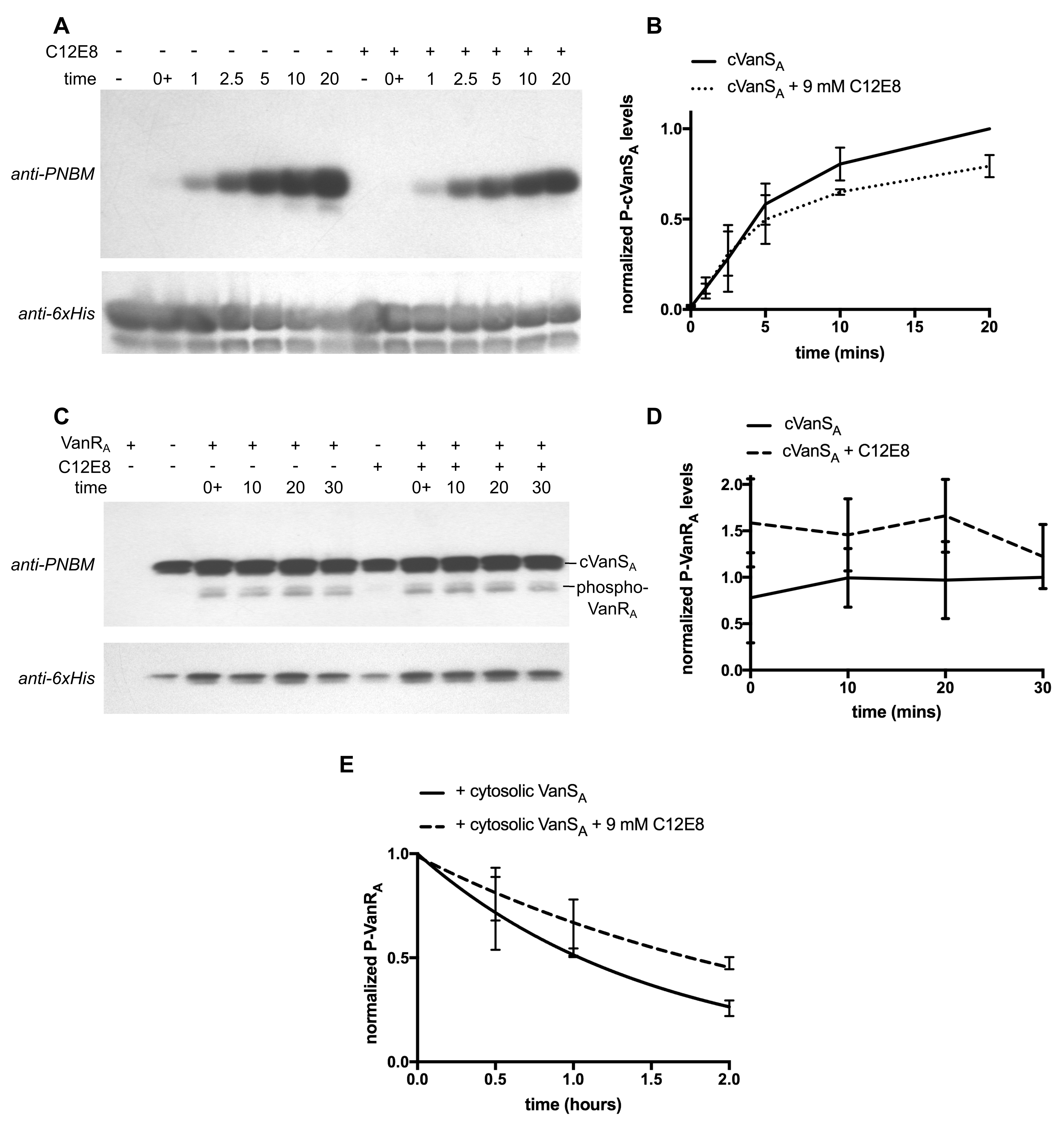

Supplement: S3 Fig — (A) C12E8 has at most a modest effect on autophosphorylation. Upper panel shows an anti-PNBM blot labeling phosphorylated cVanSA in the presence and absence of 9 mM C12E8. The anti-6xHis blot is used as a loading control. The quantitation of the autophosphorylation is shown in panel (B); band intensities are normalized to the intensity of the 20-minute time point for the detergent-free reaction. (C) Phosphotransfer is not significantly affected by the presence of C12E8. Upper panel shows an anti-PNBM blot in which both phosphorylated cVanSA and VanRA are labeled; lower panel shows an anti-6xHis blot serving as a loading control for His6-tagged cVanSA. (D) Quantitation plot for phosphotransfer reaction; band intensities for phospho-VanRA are normalized to the phospho-VanRA level at 30 minutes produced by cytosolic VanSA in the absence of C12E8. (E) Effect of C12E8 on the rate of cVanSA-catalyzed dephosphorylation of phospho-VanRA. Here we show the data from the average of 3 experiments and the fitted half-life curves for dephosphorylation with and without 9 mM C12E8. A modest reduction of activity is seen at the 2-hour time point (p < 0.05), but not at earlier time points. (TIF) [file pone.0210627.s004.tif]

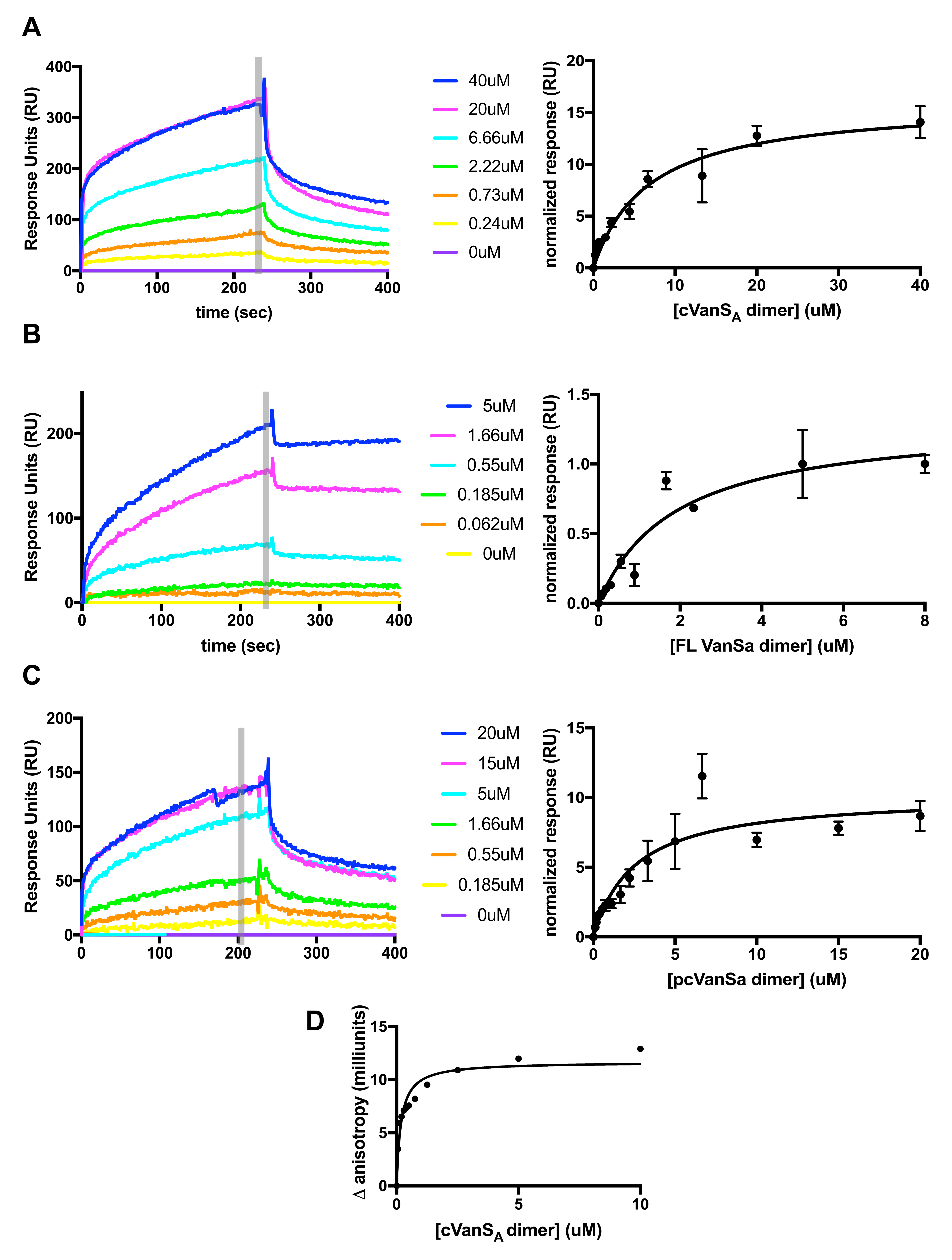

Supplement: S4 Fig — Panels (A) through (C): At left are shown representative sensorgrams from SPR experiments using immobilized VanRA. The analytes used were as follows: (A) cVanSA, (B) full-length VanSA, and (C) autophosphorylated cVanSA. The corresponding normalized equilibrium response fits are shown at right. Concentrations shown are for dimers of the histidine kinases. Gray boxes in the sensograms represent the response range used to determine the equilibrium fit. (D) Verification of VanRA binding by cVanSA using fluorescence anisotropy. A representative binding curve is shown for cVanSA binding to fluorescently labeled VanRA. The overall change in anisotropy is small, as expected for the binding of a medium-sized protein such as VanRA to a medium-sized partner. However, the binding experiments yielded reproducible results with each fresh preparation of fluorescein-labeled VanRA. The curve shown represents a binding isotherm corresponding to a KD values of 0.2 μM. Experiments done on different days with different preparations of fluorescently labeled VanRA consistently gave KD values in the range of 0.1 to 1.0 μM. (TIF) [file pone.0210627.s005.tif]

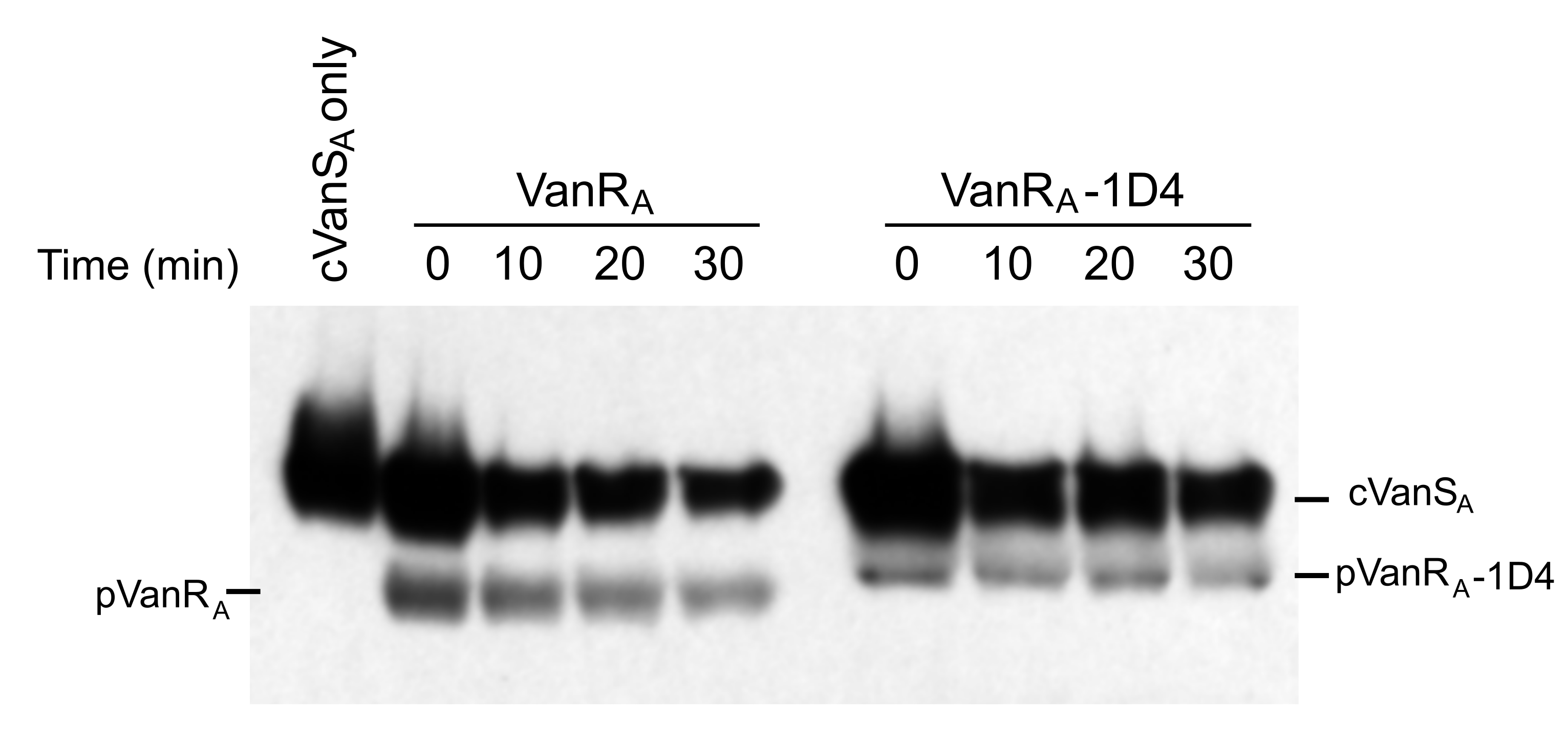

Supplement: S5 Fig — An anti-PNBM blot shows the time course of phosphotransfer from cytosolic VanSA to wild-type VanRA (left) and 1D4-tagged VanRA (right). For both VanRA constructs, rapid phosphotransfer is observed (within the mixing time of the experiment), followed by gradual loss of signal due to the phosphatase activity of VanSA. The 1D4-tagged construct migrates at a slightly larger molecular weight than the wild-type VanRA protein, owing to the additional 13 residues contributed by the linker and epitope tag. (TIF) [file pone.0210627.s006.tif]
